# Exploring the Experiences of Family Caregivers of Children With Special Health Care Needs to Inform the Design of Digital Health Systems: Formative Qualitative Study

**DOI:** 10.2196/28895

**Published:** 2022-01-05

**Authors:** Ryan Tennant, Sana Allana, Kate Mercer, Catherine M Burns

**Affiliations:** 1 Department of Systems Design Engineering Faculty of Engineering University of Waterloo Waterloo, ON Canada; 2 Library University of Waterloo Waterloo, ON Canada

**Keywords:** children, caregiver, digital health, home care, qualitative research, technology

## Abstract

**Background:**

Family caregivers of children with special health care needs (CSHCN) are responsible for managing and communicating information regarding their child’s health in their homes. Although family caregivers currently capture information through nondigital methods, digital health care applications are a promising solution for supporting the standardization of information management in complex home care across their child’s health care team. However, family caregivers continue to use paper-based methods where the adoption of digital health care tools is low. With the rise in home care for children with complex health care needs, it is important to understand the caregiving work domain to inform the design of technologies that support child safety in the home.

**Objective:**

The aim of this study is to explore how family caregivers navigate information management and communication in complex home care for CSHCN.

**Methods:**

This research is part of a broader study to explore caregivers’ perspectives on integrating and designing digital health care tools for complex home care. The broader study included interviews and surveys about designing a voice user interface to support home care. This formative study explored semistructured interview data with family caregivers of CSHCN about their home care situations. Inductive thematic analysis was used to analyze the information management and communication processes.

**Results:**

We collected data from 7 family caregivers in North America and identified 5 themes. First, family caregivers were *continuously learning to provide care*. They were also *updating the caregiver team* on their child’s status and *teaching caregivers about their care situation*. As caregiving teams grew, they found themselves working on *communicating with their children’s educators*. Beyond the scope of managing their child’s health information, family caregivers also *navigated bureaucratic processes* for their child’s home care.

**Conclusions:**

Family caregivers’ experiences of caring for CSHCN differ contextually and evolve as their child’s condition changes and they grow toward adulthood. Family caregivers recorded information using paper-based tools, which did not sufficiently support information management. They also experienced significant pressure in summarizing information and coordinating 2-way communication about the details of their child’s health with caregivers. The design of digital health care systems and tools for complex home care may improve care coordination if they provide an intuitive method for information interaction and significant utility by delivering situation-specific insights and adapting to unique and dynamic home care environments. Although these findings provide a foundational understanding, there is an opportunity for further research to generalize the findings.

## Introduction

### Background

Caring for children with special health care needs (CSHCN) in a home environment involves several complex processes, significant use of health care services, dependence on medical technology, and increased responsibilities for sharing information by family caregivers [1]. Mapping the interconnections for the most complex CSHCN reveals multilayer interactions and operations between several systems and subsystems of the health care system, all of which encompass the family caregiver and their child. These systems include but are not limited to the child’s medical teams, physical and psychological development teams, diagnostics teams, educational teams, and other medical support [1].

Home care is considered an ideal environment for CSHCN [1,2]. In hospitals, the risk of errors and adverse events is significant for children with complex health needs who require support through medical technology, enteral feeding, complex medication regimens, and mental health services [2]. However, the available technologies, services, and policies designed to assist family caregivers in coordinating care in a child’s home do not currently meet their needs [1,3]. Nondigital documentation methods that caregivers use create increasing amounts of physical health data in the home, potentially leading to errors, adverse events, and rehospitalizations because of the communication challenges and information management limitations associated with manual record keeping [4].

Although the literature identifies a need for developing electronic health records (EHRs) to organize, integrate, and communicate health information in complex home care [1], the development of EHRs remains a fundamental challenge for complex home care [5-10]. Paper-based records continue to be used as there are no substantial digital technologies available that are flexible, reliable, and trusted [11-13]. In the context of interoperability in health care, the goal is to *provide information where and when it is required* [12]. However, when billions of documents are still being created on paper, it becomes challenging to collaborate in real time, find information, and analyze and understand its meaning [12].

Personal EHR applications and web-based portals connected to hospitals or home care agencies can improve the organization and communication of health information across caregiver teams [14]. Unfortunately, caregiver engagement with these technologies is low. The annual adoption rates for web-based portals range from 5% to 12.4% [14,15]. The digital health care technologies currently being studied do not encompass caregivers’ complete information needs [16-18]. In addition, interacting with these tools is often associated with challenges that involve visual hierarchies of information, increasing the interaction burden [11]. With limited engagement in emerging digital health care tools to support home care, caregivers do not experience the potential benefits of improving home-based health management and communication [14,19].

### Study Objective

Understanding the complex work domain of family caregivers who provide home care services is critically important, given the increasing life spans of CSHCN and the growing prevalence of home care [20,21]. Few studies have investigated the potential engagement and impact of novel home care technologies for supporting caregivers. There is also a limited evidence-based understanding of how family caregivers navigate information and develop their management and communication processes. To support the design and refinement of digital health tools that can be integrated to intentionally facilitate better communication, improve the sharing of health-focused information, and ultimately contribute to improving home care, this study captures the diverse experiences and perspectives of family caregivers of CSHCN.

## Methods

### Study Design

This research is part of a broader mixed methods study on the design of digital technology to support caregivers in complex home care, which involves interviews, surveys, and a modified Wizard-of-Oz interaction [22]. In the modified Wizard-of-Oz interaction, participants listened to and provided their perspectives on prerecorded audio examples of someone interacting with different designs of a voice user interface in a home care context. The focus of this paper is to address the gap in understanding the experiences of family caregivers of CSHCN in managing and communicating health information in their homes. We conducted qualitative, semistructured interviews guided by the principles of storytelling in narrative medicine to enhance knowledge and used inductive thematic analysis to analyze the interview data [23,24].

This study was conducted remotely in North America. This research received ethics approval from the University of Waterloo research ethics committee.

### Eligibility Criteria

Eligible participants were aged ≥18 years and were family caregivers for CSHCN in their homes in North America. In the context of this study, CSHCN included children who had any combination of the following: chronic conditions, mental health issues, medication-related problems, and social vulnerability. A family caregiver was anyone who provided or coordinated care for CSHCN; they assisted the child with medication, feeding, medical treatments, medical technology use, or other health-related tasks in the home.

### Recruitment

COVID-19 restrictions led to fully web-based recruitment between June 28, 2020, and September 25, 2020. We contacted hospitals and home health care and caregiver support agencies or groups via email and social media platforms such as Twitter and Facebook, and then snowball sampling was performed on existing contacts. Study participants were sent a thank you letter for their participation after the study, and no remuneration was given for participation.

### Data Collection

A total of 2 researchers (RT and KM) conducted the interviews. Microsoft Teams was used to record the interviews, and only audio recordings were used for transcription. One of the interviewing researchers was an experienced interviewer and qualitative researcher (KM), and the other was an MASc candidate in systems design engineering with prior experience in conducting interviews (RT). After each interview, field notes were completed. There were 2 parts to this exploratory qualitative study: caregiver demographics and caregiver work domain. Participants were interviewed over video from their homes, where they provided care for their children. The first part of the interview focused on understanding the caregivers’ backgrounds and their home care situations. The second part of the interview captured the caregivers’ work domain to manage and communicate information and care responsibilities in their homes. The interviewers asked the participants to describe how they navigate caring for someone in their home and communicate with other caregivers and the factors that influence their home care environment.

### Data Analysis

The interview data were analyzed through inductive thematic analysis using the following steps: (1) the interviews were transcribed verbatim; (2) members of the research team read the transcripts and listened to the audio recordings to familiarize themselves with the data; (3) core team members thematically coded the data; (4) initial codes and themes were developed; and (5) the data were presented to the whole team for discussion and refinement. Data were stored and organized using QSR NVivo 12 and Microsoft Excel 2021. All names and identifiers were made anonymous during the transcription process. Triangulation of the data was achieved using various geographic areas, multiple coders, and a multidisciplinary team of researchers interpreting the results. Data saturation was reached after 7 interviews when no additional themes were identified.

## Results

### Study Population

A total of 7 family caregivers of CSHCN participated in this study (Table 1). Of these 7 caregivers, 2 (29%) family caregivers participated from the United States, and 5 (71%) family caregivers participated from Canada. Of the 5 participants from Canada, 4 (80%) were from Ontario. The youngest participant in this study was aged 33 years, and the oldest was aged 40 years. All participants identified as female, and the caregivers’ experience of providing care in their home for their child ranged from 4 to 18 years. Medical equipment and tools that family caregivers operated to support their children in their homes ranged from gastrostomy or gastrojejunostomy tubes and cough assist machines to ventilators and mobility devices. Although the sample size was small, the objectives of this study were to explore the rich experiences of a diverse group of participants based on age, caregiving experience, and location, which led to the development of meaningful themes regarding information management and communication in this complex health care domain.

**Table 1:** Participant demographics and caregiving characteristics (N=7).

Characteristics		Family caregivers, n (%)
**Age (years)**		
	25-34	2 (29)
	35-44	5 (71)
**Gender**		
	Female	7 (100)
	Male	0 (0)
**Location**		
	Ontario, Canada	4 (57)
	Alberta, Canada	1 (14)
	Missouri, United States	1 (14)
	Minnesota, United States	1 (14)
**Caregiving experience (years)**		
	0-5	1 (14)
	6-10	3 (43)
	11-15	2 (29)
	16-20	1 (14)
**Medical equipment or tools**		
	Cough assist machine	1 (14)
	Gastrostomy or gastrojejunostomy tube	3 (43)
	Orthotics	2 (29)
	Oxygen concentrator	2 (29)
	Oxygen tank	1 (14)
	Percussion tool	1 (14)
	Pulse oximeter	2 (29)
	Shake vest	1 (14)
	Shunt	2 (29)
	Suction unit	2 (29)
	Ventilator	3 (43)
	Wheelchair or walker	2 (29)

### Thematic Analysis

Coding conducted by the research team led to the identification of 47 codes (Table 2). The list of codes was developed into 5 themes and 4 subthemes describing the information management and communication processes of family caregivers of CSHCN in a home care domain.

**Table 2:** Themes in communication and management of information in complex home care.

Themes and subthemes			Codes
Continuous learning to provide care			Connecting with other families Learning from health care professionals Learning from therapists and technicians Learning from training Learning about medical technology Learning by observing Figure it out on my own Navigating through information for complex children Learning what to do in emergencies Learning procedures
**Updating the caregiver team**			
	Maintaining records	Physical documentation Identifying patterns Burden of documentation Documenting vitals, health status, and medications Documenting holistic aspects of care Documenting equipment settings Desire for digital records Adapting documentation as a child’s condition changes Transparency of record keeping Concerns for information security Desire to ease record keeping	
	Sharing the right information with the right person at the right time in the right way	Posting information around the home for other caregivers Feeling pressure Financial consequences Communicating with health care professionals Ensuring situation awareness Summarizing changes Memorizing information	
	Strategizing care with the caregiver team	Sharing recent health information Troubleshooting health care issues Identifying appropriate therapies and treatments	
**Teaching caregivers about their care situation**			Teaching through documentation Requiring background knowledge of caregivers Sharing their child’s journey Share care expectations for home care tasks (eg, feeding)
	Communication challenges in teaching caregivers	Struggling with the consensus of caregiver training Effectiveness of training Trust in caregivers	
Communicating with their child’s educators			Receiving health updates from the school Creating health care tracking documents for the school
Navigating bureaucratic processes			Transferring information to governing bodies Preparing caregiver schedules Negotiating personal care hours Managing caregiver hiring Acquiring funding Documenting caregiver information Impacts of COVID-19 on home care services

### Continuous Learning to Provide Care

Family caregivers of CSHCN explained that they were continuously gathering information to learn about their child’s condition and then applying this knowledge in their home. They explained that they collected information from various sources, including trained professionals (3/7, 43%); media such as books, videos, and other documentation (4/7, 57%); by observing other caregivers (2/7, 29%); and from family caregiver networks that connected them with caregivers of children with similar conditions (2/7, 29%).

At the onset of navigating through their home care situation, Participant 1 and their caregiver team of nurses received specific medical information and training from their local children’s hospital:

As soon as we got our team hired, we were able to send them all to [the hospital] for training, which is where my husband and I had to pass a course...to basically show we could save our [child’s] life before we were discharged from the NICU.

Participant 1

However, with the uniqueness of CSHCN conditions and treatments, which can sometimes be rare and difficult to diagnose, the family caregivers in this study had varied experiences concerning the professional training or resources provided to them:

There’s only one type of parenting guidance that’s out there. [Having a] child with all these complex needs...and you are expected to just know how to navigate or facilitate your way through all of it.

Participant 4

Family caregivers who did not receive specialized training relied on their observation skills to mimic the required processes that they saw in clinics or hospitals:

With our suction machine, when we initially came home with it, the day of discharge from the hospital, I was handed the suction machine, “Here’s your machine. Go home bye.” Nobody showed me...I have never seen a user manual for those ever...At that point, I had gotten used to seeing them at the hospital...the wall-mounted suction machines. I kind of had a basic idea...Like, “OK, can somebody at least show me how to turn it on?”

Participant 5

Family caregivers who communicated on the web with caregiving networks explained that these were tools to reduce their reliance on their health care team. They used this network to discuss their concerns and work together with others whose children had been through similar experiences:

I get a lot of information from a mom’s group...You don’t necessarily want to be calling the clinic every time something comes up...unless it’s serious...So, you know, we might say, “Oh, I noticed she’s starting to get stomach aches. What has the group seen?” That kind of thing. It also helps us to try a few things before we call the clinic and say, “She’s experiencing these symptoms. We’ve already tried X, Y and Z,” which those X, Y and Z I usually get from the mom’s group, for things that they’ve gone through similarly. They’re the people who know, they’ve been there.

Participant 3

### Updating the Caregiver Team

#### Overview

Family caregivers of CSHCN are often the primary knowledge holders for information about their children [25]. In this study, each participant discussed the pressure to maintain awareness of their child’s history and current health status among all caregivers to ensure that their child received the best possible care. Their communication responsibilities are summarized with the following subthemes: *maintaining records, strategizing care with the caregiver team,* and ensuring that they share *the right information with the right person at the right time in the right way.*

#### Maintaining Records

A critical process that family caregivers carry out to update their caregiver teams is documentation. caregiver in this study implemented paper-based documentation at the start of managing their child’s condition and recorded information such as vitals and medications (5/7, 71%), the status of their child’s life-supporting equipment (2/7, 29%), or other holistic aspects such as behaviors and feelings (2/7, 29%). Of the 7 caregivers, 3 (43%) continued to record detailed information about their children in paper-based records every day, with children aged 4 to 13 years at the time of this study. The other caregivers (4/7, 57%) documented health information infrequently.

Retaining detailed information about their child’s care was a burden for the family caregivers in this study who were documenting every day, which was apparent when they described having to continuously condense and summarize this knowledge for other caregivers. For example, Participant 4 expressed the challenges associated with the amount of information that they had retained and the impact that this had on the expectation for them to remain the primary knowledge sharer in their caregiver team:

There are always people added and incoming, and it seems like it’s always on the parent to fill in the next carer, the next professional, on what’s going on and answer their questions of what they may have from the previous professional. And it all comes down to the whole concept of the parent is the expert in their child...But we shouldn’t have to be that role all the time, and that’s very frustrating...We have team meetings at the school once a month with teachers and board members and all that stuff, and I'm expected to come in with my binders of information and fill them in on everything that’s happened in the last month...It’s crazy exhausting. It was stressing me out considerably.

Participant 4

Maintaining their children’s health documentation in the home was not a responsibility that some family caregivers in this study mentioned conducting on their own. Multiple caregivers (4/7, 57%) described sharing documentation responsibilities with other caregivers and developing methods to ease their documentation processes through checklists and sign-off sheets:

I also have a binder that I, well, technically, I have three, but let’s not get carried away. I have one I call [my child’s] Bedroom Binder. And in [my child’s Bedroom Binder] is the medication schedule and a check-off or a sign-off sheet so on every day of the month there is an opportunity to sign off every medication dose that [my child] receives that day, who gave...

Participant 2

I would just have them like do a little checklist so that they wouldn’t forget to do a med or something like that.

Participant 7

However, because of the COVID-19 pandemic, there was a reduction in paper-based records and verbal communication with other caregivers. One of the caregivers expressed that they were spending more time using email to receive updates from other caregivers about their child’s condition:

[We were using paper records and having conversations in-person] until recently, and now they will email me an update...“Today we found...This is what you need to be working on...”

Participant 4

Ultimately, no participants in this study described using a digital health tool to document and track information, despite their excitement and hope toward digitizing their current processes. Only 1 participant (Participant 6) used a Google Home voice assistant to remind their child about medications and appointments. Another participant (Participant 2) explicitly expressed frustrations with a software application they tried to use to support care information management in their home. However, the associated steps involved in navigating the application and the limited customization rendered it useless:

One of the most annoying things about it is that it’s an app on my phone, and I have to sign into it every time I click on it. It pisses me off...I actually don’t use this because it doesn’t accept a couple of [my child’s] diagnoses. It doesn’t recognize them. It doesn’t recognize some of [their] medications, and it has rendered itself useless because these are [the] things we do every day...I had pretty high hopes and was really excited. And I’m just really frustrated and disappointed that it’s not what I wanted it to be.

Participant 2

#### Strategizing Care With the Caregiver Team

Along with providing care, participants described that their caregiver teams were also responsible for developing and implementing strategies to improve care quality in their homes. Capturing their child’s health information was a critical step in the process of strategizing care, where many family caregivers in this study (5/7, 71%) explained that they provided their caregiving teams with the necessary details for identifying correlations and patterns in their child’s health:

When [my child] sees the complex care team at [the children’s hospital], we’ll take the chart with us if we need [the] clinicians to troubleshoot something with us.

Participant 1

Between school and home, and for behaviours and possible seizures, we were recording [them] so we could take it to the doctors...I wasn’t there [at school], and I was just trying to go by [what their educators were saying].

Participant 6

#### Sharing the Right Information With the Right Person at the Right Time in the Right Way

A factor related to the documentation and communication of health information about their child was sharing the correct information with the right person at the right time in the right way. The caregivers in this study expressed the cognitive demands and consequences associated with properly filtering large amounts of information wherein they had to ensure that they effectively shared the necessary details with those who needed it:

I think that remembering to share the right information with the right person at the right time in the right way so that they hear what they need to hear so that they will be willing to help us is the precipice of my existence...I feel that pressure in every conversation I have about [them].

Participant 2

The financial consequences of remembering to share specific details with the right person at the right time also affected family caregivers’ stress (2/7, 29%):

If we go into an appointment with a physical medicine specialist and I forget to tell [them] that [my child’s] got really good range of motion in [their] feet, so [they] only needs rigid [ankle foot orthotics (AFOs)]...I can miss getting that prescription for AFOs. And without that prescription, I can’t get them covered or made because vendors in this area won’t even make you a pair of AFOs if you don’t have government funding.

Participant 2

### Teaching Caregivers About Their Care Situation

#### Overview

Information management and health communication played an essential role for family caregivers who discussed teaching and training caregivers about their home environments, care plans, and specialized medical technology, which took time away from their child’s care (6/7, 87%). Participants expressed that they needed a range of 3 to 80 hours to train each new caregiver. Participant 2 explained that they often needed to train new caregivers every 6 months.

To support caregiving education and ensure that the caregivers in their home understood the nuances of their child’s care needs, the family caregivers in this study developed their child’s health care information into physical teaching materials, documentation, and training methods. Among several binders that Participant 2 created in their home, 1 binder was specific to teaching other caregivers how to communicate with their child effectively:

[The] binder also has stuff about basic communication with [my child], and I have developed what I call a gesture dictionary...I start [my hired caregivers] with that piece right away: “You need to read this. You need to reference it when you can’t figure out what [they’re] telling you. This is really important.”

Participant 2

Along with physical documentation, the family caregivers in this study also relied on in-person training to communicate their home care’s subtle nuances:

Picking up on [my child’s] little signs that [they do], that’s where...I have to tell them cause...that’s kind of hard to have it written down. It really is a show-and-tell...you have to hear it to understand it.

Participant 5

And it’s trying to teach the workers how to tell the difference between “I don’t want to” versus “I can’t.”

Participant 6

Although sharing the information related to their child’s care and the specific processes involved was one aspect of their training, one of the caregivers specifically expressed the additional importance of communicating their child’s growth:

The information I always wanted to share is where [my child] has come from...The fact that [they were] in a vegetative state for the longest time, [they] couldn’t walk, [they] couldn’t talk, [they] couldn’t do anything and now [they run], [they do] track and field, [they play] basketball, [they] can talk...[They] can do a lot of stuff...I think it’s important for people to always know where you’ve been.

Participant 6

#### Communication Challenges in Teaching Caregivers

Despite the family caregivers’ resiliency in this study, they still experienced communication challenges in their roles as caregiving educators (3/7, 43%). This was especially evident when participants expressed the challenge of training caregivers who already had a knowledge base and their own best practices:

Someone who has a willing heart, and mind, and a desire to look upon our world is easier to train than [a health care assistant] or [licensed practical nurse] that’s worked in the field, in institutions, for ten plus years because they’ve got patterns and rhythms and things that are important to them that I’m not really bloody interested in having in my home.

Participant 2

Participant 6 described the challenge of their hired caregivers being receptive to the training and their child’s specific needs:

...and the information that you’re willing to accept, right? Like I can train you on how to do a transfer 100 times, but if you’re not receptive to the training, you’re going to do it the way you want to.

Participant 6

Training caregivers was especially challenging in the context of the COVID-19 pandemic. For example, Participant 2 expressed challenges in communicating with their hired caregivers about proper mask wearing in their home:

I have a really hard time right now in this whole COVID situation. Getting the girls that come into my home to wear their masks effectively and appropriately...The biggest problem I have is that they are always touching their face, and it’s like, every time you touch your mask on your face, you have to wash your hands.

Participant 2

The effectiveness of their training was an additional concern for the participants in this study. They expressed uncertainty about whether their caregivers applied their training knowledge appropriately to provide care for their children:

I have several women that work for me who have no medical background at all...I can explain to them why I want them to do this, and they don’t really get it. They don’t understand contamination...And I don’t know how to effectively explain that to people and get them to work through it. That’s really difficult.

Participant 2

Their inability to trust that their training was being implemented was one reason why Participant 7 no longer hired caregivers:

I cannot trust people to do things or do it right or make the right decision. And even if they can’t make the right decision...just knowing to call me. Some of the times where they brought [my child] to the hospital when [they] didn’t need to go to the hospital, and they brought [them] to the wrong hospital rather than just calling me and asking...

Participant 7

### Communicating With Educators

Family caregivers of CSHCN attending school often had the additional challenge of navigating communication methods and information management with their child’s educators (3/7, 43%). Sharing information with their child’s school was done through verbal communication in person or by phone and written notes or email. Some family caregivers in this study experienced difficulties in receiving valuable information to track how their child was developing to continue building on this development at home:

Who [my child is] at school is...very different...than who [they are] at home...We need to know what’s going on there so we can mimic here...How did we get to this year with this many children who have needs and their people still don’t understand how to do these communications.

Participant 4

The technologies used to support the sharing of information among their child’s educators were not consistent. Caregivers described different methods that educators used to collect and transfer information about their child, which raised concerns about their child’s safety:

It’s a verbal chat, or it’s an email or...we have been using emails more often because I don’t answer my phone anymore...I like to have things written down. Or there’s like a scrap piece of paper in [their] lunch pail or something...I do know now with the different portals, and stuff that different doctors have would have been easier in the moment...I had three adults that were being paid...[my child’s] school supports...videotaping [my child] and walking around with that on their cell phone. So, there’s no security or confidence that...it just opened up a whole can of worms. Or it had a potential of opening up a whole can of worms, but it’s all we had...If there were secure ways of doing it, it would have been a little bit safer.

Participant 6

In other situations, the family caregivers in this study (2/7, 29%) requested that their child’s educators continue to track their child’s health care metrics using their personally designed tracking sheets while being cognizant of workload:

You would think—and there’s only like four to six kids in [their] class—you would think that [their] teacher would be able to like ﬁll out a quick form...but she never did it. So, then I realized like “OK, maybe it’s because she feels like it’s too much work,” so I altered it to just be...kind of the general just circle it...So, the more people have to ﬁll out, the less likely they are to do it. That’s what I’ve realized...People are lazy, is what I’ve learned. [laughs] Even the really good ones. She’s a fabulous woman, but like they’re still lazy.

Participant 7

Another family caregiver used a communication book; however, despite their child’s educators writing in them, the information did not provide insight into their child’s care or development:

[Their] communication book would come back, and it would be like, “today [they] had a great day.”

Participant 4

### Navigating Bureaucratic Processes

Although not directly related to health information management, navigating the management of their child’s information with the bureaucracy of home care was a process that added another level of complexity, which was expressed by some participants in this study (2/7, 29%). The participants expressed their responsibility to organize the necessary paperwork to have hired caregivers, such as scheduling, timesheets, and payroll:

The main criticism of the family-managed homecare program is the amount of paperwork that’s required of families. But now that we’ve been doing this for two years, between my husband and I, it may take two to three hours a month to do payroll and the paperwork.

Participant 1

Payroll is something that I have kind of hired out, so I have to make the schedule and tally the timesheets, but then I have a really lovely company that I've been interacting with since day one, and they have saved my butt more times than I can count, and they charge me a nominal [fee], and they do all of my EI and CPP, and they interact with the CRA on my behalf. They interact with WCB on my behalf. I send them signed checks. This is how much I trust this company, and they have never done anything wrong. If anything, they have saved my butt.

Participant 2

The impact of the COVID-19 pandemic further influenced the ability of participants to acquire home care support and services, requiring them to manage more aspects of their child’s health information and communication on their own. The participants in this study reported having reduced support because of public health restrictions or their own choice—to limit their child’s exposure—and the pressing need for home care solutions in the era of the COVID-19 pandemic:

So we actually lost all of our care...I would say 98% of the families out there in Ontario lost all of their services when the COVID lockdown happened because they were not deemed essential...We went from having 11 hours of support a week to having zero.

Participant 6

I know that families that I speak with, we’re all struggling to find ways to get support without being able to bring in support.

Participant 4

## Discussion

### Principal Findings

This exploratory study examines how family caregivers navigate information and the processes involved in health communication in complex home care to support the designing of digital health information systems. The family caregivers of CSHCN in this study were underserved concerning the tools available to support them in managing their child’s health care in their homes [11]. Given that children with medical complexities account for one-third of all health care spending on child health in some North American regions, it is critical to understand their experiences to inform the design of digital information management tools that can improve home care [26].

### Critically Important Contexts of Home Care Delivery

Although recognizing that all CSHCN are fundamentally unique [27,28], the context of family caregivers’ home care situations plays a critical role in information management and communication experiences for CSHCN. The social determinants of health have been shown in prior literature to influence inequities in health care delivery [29]. There is also a need to use digital health tools to better account for these inequalities in our health care system in clinical settings [29]. As observed in this study, the design of digital health technology needs to consider supporting the context of home care. For example, for the participants in this study, information support provided by the health care system was either easily accessible or required significant work on behalf of the family caregiver to be accessed. Opportunities for formally trained hired caregivers were also readily available or nonexistent. Life-supporting medical equipment is often a significant component of complex care for families of CSHCN [27,30]. However, some families in this study were also not able to access the necessary instructions or formal training and took on considerable responsibility to ensure their child received safe and high-quality care by gathering information on their own and developing standard care practices for their home. Schaepe et al [31] argue that family caregivers are necessary and provide value and knowledge to their child’s home care. However, there are significant risks to patient safety in life-threatening situations if family caregivers are not formally provided access to the required knowledge [31]. A recommendation provided by Foster et al [32] was to improve home care policies for families of children with medical complexities by including home health training through partnerships with pediatric health care systems. In the context of digital health tools, there is potential to design information technology that can provide accessible health care knowledge and training information. Universal access to information on the web or through mobile platforms can address inequalities for families who otherwise do not have the means to safely coordinate home care and provide health care services for their children with complex conditions.

In the context of the social attributes for providing home care with respect to caregiving stress and the support for caregiving services, some family caregivers shared specific issues around having access to medically trained hired caregivers for respite care. Keilty et al [33] identified severe consequences for family caregivers of a child with a medical complexity who experience sleep disturbance, which may be relieved by addressing respite needs. Although some participants in this study eventually received access to professionally trained caregivers to support their 24-hour home care, others navigated the hiring process and medical training and supervised hired caregivers independently. One may argue that these processes mirror those of a small business or full-time job [34,35]. The challenge with receiving respite care in this study was either because of the limited availability of professionally trained caregivers from local agencies or geographic location, specifically when families live in rural regions where local agencies do not exist, as identified by Weaver et al [36]. With digital technologies disrupting conventional business models and delivery services, especially during the COVID-19 pandemic [37], digital health tools have increasing potential to support respite care by making connections with hired caregivers a more effective and efficient experience for family caregivers of CSHCN.

The additional challenge of training a stranger with no medical experience places considerable pressure on family caregivers who may not be in a position where they have the time, resources, and overall capacity. They simultaneously maintain the health and safety of their children while training someone about their developed methods and processes for performing care. Respite care for the family caregiver may be minimal while effectively training someone with no medical experience. As this study identified, training a new caregiver may require up to 80 hours through several 1- to 8-hour shifts before the family caregiver and trainee are comfortable caring for the child independently. This study further identified that the resources provided to support training are often scarce. When they are available, the family caregiver is responsible for sharing the materials in an organized manner to support effective and efficient learning. With the compounding responsibilities for providing care, many family caregivers quit their full-time jobs to stay at home and provide for their child’s health care needs on their own [27].

### The Evolutionary Home Care Complexities for Family Caregivers of CSHCN

Information management and communication processes are dynamic for family caregivers of CSHCN [38]. However, this study identified that family caregivers remain steadfast users of paper-based systems, which do not entirely support the needs of their caregiving tasks. As their child grows older, the types of support they need change, and new caregivers enter and exit their child’s caregiver team. In addition, caregivers continuously learn new information about their child’s health conditions. As a result, the information they track may change from recording vitals when first providing care to recording holistic aspects of care such as behaviors and feelings. They also design alternate versions of these forms for other caregivers to use. In some situations, family caregivers may record information less frequently. The family caregiver may only need to track information if their child is experiencing unusual symptoms to identify the underlying patterns and trends and report them to their clinician team in an email. Email messaging does not provide a standardized or secure method for effectively communicating health information. In addition, the paper-based methods that the caregivers used in this study to support dynamic processes needed to be continuously adapted by the family caregiver by updating their nonstandardized record-keeping templates created on a computer or by designing and printing new templates.

Although documentation processes may change or reduce in frequency based on their child’s evolving health care needs, new challenges emerge for navigating 2-way communication of their child’s care while attending school, which has also been identified by Mikles et al [39]. Although educators are often not trained medical professionals or health communicators, they can be considered a part of the caregiver team when they become responsible for supporting CSHCN in their classrooms. The caregivers in this study currently use technologies such as email, voicemail, and written notes to meet information exchange methods between their homes and their children’s schools. No caregiver in this study described using digital health applications designed to support health information management and communication. With the technologies they were using, mainly including paper-based documents or keeping information on photograph and video storage applications on others’ devices, privacy concerns exist for misplacing sensitive information or for individuals to have unsecure access to information.

Nonstandardized methods of communication that do not follow defined protocols for the type of information that needs to be shared are also associated with challenges. These challenges include communicating health insights that provide value toward improving the child’s care and facilitating the organization of this information for efficient access by caregivers in the future. Although technology supports, such as web-based portals, have been developed to securely communicate health information from hospitals and other care facilities, and in some situations, there are standard physical documentation and handoff protocols that can be provided to a family to support the communication of a child’s development in school [39], the general needs of family caregivers of CSHCN to facilitate 2-way communication with their child’s educators have not been considered in the design of digital technologies.

### Design Recommendations for Digital Health Care Technology

**Figure 1:**
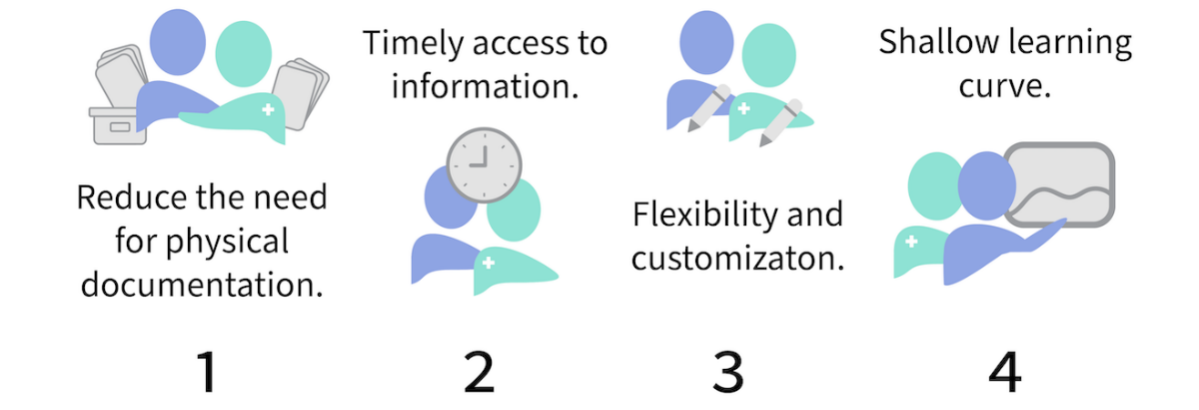
Design recommendations for digital health tools in complex home care.

This study identified a significant cognitive and time burden for family caregivers of CSHCN to share complete information accurately and concisely regarding their child’s health with other caregivers [40-42]. Information sharing occurs within caregiver handoffs in the home, at caregiver team meetings in clinics and schools, and with their child’s physicians and specialists [39]. Digital health tools have a significant potential to reduce the pressure on family caregivers while managing their children’s information [11,43,44]. With the work that family caregivers currently perform to overcome challenges related to the lack of caregiving support and resources, it is critical to design digital tools to support natural interactions while providing significant utility. The design recommendations are shown in Figure 1.

First, the focus should be directed toward designs that reduce the need for family caregivers to physically organize their collected information manually, preventing accidental duplication and reducing workload. Many of the current tools available to caregivers require laborious data entry, are rigid systems that do not permit customization, and do not link health care professionals or other caregivers in real time [11,45]. A similar recommendation has been made in the context of health insurance tracking for families of CSHCN [46]. Although systems to automatically monitor health conditions exist, such as wearable devices (eg, Apple Watch, Fitbit, Garmin, and Polar) or other ambient, noncontact tracking systems [47,48], the complexity and uniqueness of home care for CSHCN with respect to the child and the data that is required to track may not always be suited to the use of these technologies. There may also be other barriers that prevent the introduction of these technologies to family caregivers in their homes. As a potential solution, voice interaction technologies to collect spoken health metrics or other information may be an alternative to enabling caregivers to track data without requiring them or their child to physically interact with a device [11]. Although the spoken information may be unstructured, the system could be designed to identify keywords, intents, and measures to enable practical data storage and information retrieval in a structured database.

Second, digital health tools should provide timely access to information, insights, and patterns specific to the context of the caregivers among whom the information needs to be shared. With the cognitive burden that family caregivers face in accurately sharing information with others, digital tools should support caregivers to access information efficiently and effectively depending on with whom they are interacting and their current working environment. For example, in the clinical setting, design recommendations for digital health technology development to support health care management of pediatric blood marrow transplant patients have been described by Shin et al [42]. Although not explicitly describing how the user would interact with the technology to access insights about the health data, their results similarly point toward the idea of a digital health tool that provides general use in tracking patient symptoms, consult visits, and medications, supporting the caregiver’s cognitive load to remember details accurately [42]. Shin et al [42] further recommend providing overviews of the visit to the caregivers in a manner that promotes an accessible understanding of the information to support an engaging clinic visit. Previous recommendations have also been made to design digital health technologies that facilitate document sharing among providers, family caregivers, and educators [45] and automatically send new information to the care team individuals requiring it [39]. To build on these recommendations, developing digital health technologies that center the family caregiver’s needs to ensure that the right person receives the correct information at the right time should be prioritized to alleviate cognitive burdens.

Third, digital health tools should allow for flexibility and customization regarding the inputs and outputs of the display to meet the needs of complex home care over time. Adaptation is essential in complex home care when a child’s health condition constantly changes over time. In the closely related caregiving domain of child development in educational institutions, similar emerging recommendations have been made—to design digital health technology that supports adaptation for continuously changing caregiving information and communication needs [39]. Recommendations from Mikles et al [39] point toward configurable patient referral reports that provide customization relevant to the relevant stakeholders. The researchers also recommend the inclusion of test results, descriptions and notes, medications, languages, race and ethnicity, care summaries, and health summaries [39].

Finally, any developed digital health tool should offer a shallow learning curve to promote a more intuitive interaction for all caregivers who need to interact with the information. Family caregivers already spend considerable time learning about their children’s conditions and the complex medical technologies and medication regimes they require [3,27]. Providing a tool that provides a natural interaction could reduce the learning curve required to use digital health tools and support future engagement by a wider group of users [11]. As a positive consequence, digital health tools that are easier to interact with may subsequently be used as an additional channel to support family caregivers in learning about the conditions of their child and their medical devices in a more effective manner [49].

### Strengths and Limitations

This exploratory study captures the emerging perspectives of family caregivers of CSHCN from a diverse group of participants, including wide-ranging home care contexts and caregiving experiences to represent breadth in caregiving knowledge. Although this research is not explicitly focused on the impact of the COVID-19 pandemic, it did occur during the pandemic, giving important consideration to times when caregiving practice has had to shift rapidly, which is another under-researched area. Caregiver perspectives, the stresses that they have experienced, and their capacity to manage and communicate health information in their homes may have been influenced by the ongoing changes resulting from the pandemic, and there is an opportunity for future research to examine this further.

The participant demographics are limited because of the exploratory nature and practicalities of conducting this study during the COVID-19 pandemic, which affected recruitment because of the time limitations of potential participants caring for children who are medically complex who often require care or other support at all hours. Furthermore, although this study reports on geographical locations and other distinct demographic data for transparency of the sample population, only 2 caregivers were included from the United States. However, this study is guided by the principles of narrative medicine storytelling, where including their data introduces critical perspectives that may identify avenues for future research [24]. Although the data analysis reached saturation, allowing for the development of meaningful themes that provided a rich understanding of the experiences of the family caregivers in this study, a larger sample size would support generalizing these findings to a wider caregiving population. Future work will build on this with broader demographics that include insights on the impacts of caregivers’ financial situations while examining differences between health care systems in Canada and the United States that can inform design. There is also the potential to examine this population more deeply for specific diagnoses and long-term implications of changes in care.

### Conclusions

Our formative research study provides a foundation for some of the emerging challenges related to family caregivers’ information management, communication, and caregiving support in complex home care. Furthermore, it begins to identify opportunities for digital health tools to support gaps in the health care system. Digital health solutions may address unmet caregiving needs for access to medical device information and training material, shared situational awareness with other caregivers, and access to caregiving services, including respite care. With the complex information processes that family caregivers of CSHCN are involved in daily, high-level design recommendations for developing future digital health technologies point toward solutions that facilitate intuitive interactions while providing utility through timely access to organized, context-specific data.

Digital health tools for complex home care may also improve the cognitive burden associated with the health care tasks involved with being a family caregiver of a child with complex health care needs—solutions that may lead to safer coordination of care. As digital health tools continue to be developed, future research should focus on designing digital health care tools in close collaboration with the multiple stakeholders involved in care, including diverse family caregivers. Ultimately, the preliminary findings from our study may provide valuable insights for informing the design of digital information management and communication systems in complex home care for family caregivers of CSHCN.

## Abbreviations

CSHCN: children with special health care needs
EHR: electronic health record
